# A randomized trial of the effects of flaxseed to manage constipation, weight, glycemia, and lipids in constipated patients with type 2 diabetes

**DOI:** 10.1186/s12986-018-0273-z

**Published:** 2018-05-09

**Authors:** Noureddin Soltanian, Mohsen Janghorbani

**Affiliations:** Isfahan Endocrine and Metabolism Research Center, Isfahan University of Medical Sciences, Isfahan, Iran

**Keywords:** Flaxseed, Efficacy, Constipation, Diabetes, Lipid, Glucose

## Abstract

**Background:**

To compare the effects of baked flaxseed versus those who received a placebo on constipation symptom scores, weight, glycemic and lipid control in constipated patients with type 2 diabetes (T2D).

**Methods:**

In a single-blinded, randomized controlled trial, 53 constipated patients with T2D with body mass index (BMI) 20.5–48.9 kg/m^2^ received either 10 g of flaxseed pre-mixed in cookies twice per day or placebo cookies for 12 weeks. The constipation symptom scores, BMI, fasting plasma glucose (FPG), glycosylated hemoglobin (HbA1c), and lipid profile were determined at the beginning and end of 4, 8, and 12-week period. Constipation was evaluated with a stool diary (ROME III).

**Results:**

After the 12-week intervention, constipation symptom scores (2.46), weight (− 3.8 kg), BMI (− 1.5 kg/m^2^), FPG (− 26.7 mg/dl), cholesterol (− 37.3 mg/dl), triglycerides (− 10.4 mg/dl), LDLC (− 21.0 mg/dl), HDLC (4.7 mg/dl), cholesterol/ HDLC ratio (− 1.4 mg/dl) significantly decreased from baseline in the flaxseed group (all *P*-values < 0.05). The differences of absolute change of constipation symptom scores (2.46 vs. 0.41), weight (− 3.8 vs. 0.0 kg), BMI (− 1.5 vs.-0.1 kg/m^2^), FPG (− 26.7 vs.-1.9 mg/dl), >HbA1c (− 0.8 vs. 1.0%), cholesterol (− 37.3 vs. -10.4 mg/dl), LDLC (− 21.0 vs. -4.3 mg/dl), and HDLC (4.7 vs. -4.4 mg/dl) between the flaxseed and placebo groups were statistically significant (all *P*-values < 0.05). The compliance was good and no adverse effects were observed.

**Conclusion:**

In constipated patients with T2D, flaxseed cookies used as a snack may be a useful tool for decreasing constipation symptoms, weight, glycemic and lipid levels.

**Trial registration:**

irct.ir: IRCT20110416006202N2.

## Background

Type 2 diabetes (T2D) is an increasingly common chronic disease and a major health concern that has been expected to continue far into the future [1]. Constipation is one of the complications seen in patients with T2D and its prevalence has been shown to be higher in patients with T2D than in the general population [2]. Although constipation is a predictable complication of T2D, inadequate management of constipation is common and may have profound implications [3, 4]. Its treatment remains challenging because most of the patients are not satisfied with current therapies due to lack of efficacy or safety or availability [5]. Compelling evidence supports the role of diet in the management of T2D. A meta-analyses found that flaxseed intervention improved lipid profiles and reduced CVD risk [6]. Although a few studies have been evaluated the health benefits of flaxseed in patients with T2D and indicate that it may be improves glycemic and lipid control [6–10], bowel movement [9], and body weight [7], no data are available for the constipated patients with T2D. Therefore, it is reasonable to examine evidence of clinically meaningful health benefit before selecting or recommending a flaxseed supplement to patients being treated for T2D and chronic constipation.

Flaxseed (linseed) is a functional food that is a rich source of polyunsaturated fatty acid, mainly alpha linolenic acid, an omega-3 fatty acid, as well as soluble fiber, lignan precursors, and other substances that may have health benefits, whose mechanism of action has remained elusive [11, 12]. However, the effects of flaxseed on T2D and chronic constipation remain unclear.

Therefore, this single-blinded, randomized, placebo-controlled trial was designed to test the hypothesis that the 10 g flaxseed consumption per day, as compared to a placebo, would reduce constipation symptoms, body weight and improve the glycemic and lipid levels in constipated patients with T2D. The objective of this trial was to assess the beneficial effects of adding flaxseed to the normal diet for 12 weeks of intervention among patients with T2D and chronic constipation.

## Methods

The study was approved by the Isfahan University of Medical Sciences ethics committee (approval no. IR.MUI.REC.1396.3.464), and was conducted in accordance with Good Clinical practice. The study protocol was registered at irct.ir as IRCT20110416006202N2.

### Patients and trial design

This is a single-blinded, parallel-design, randomized, placebo-controlled trial, conducted for 12 weeks. Fifty-four consecutive constipated patients with T2D attending outpatient clinics in Isfahan Endocrine and Metabolism Research Center affiliated to Isfahan University of Medical Sciences, Iran from 20 Jan. to 15 Oct. 2017 were included. Constipation was diagnosed by the Rome III criteria [13]. A one-week baseline placebo phase, where patients were not allowed any laxative treatment, preceded a 12-week treatment phase, and followed by 4-week no treatment phase. Patients were included if they had a bowel movement frequency of < 3/week during the past 3 months [13]; age ≥ 30 years, and diabetes duration > 3 year. Patients were excluded from the study if they had type 1 diabetes, weight loss, use of lipid-lowering drugs, fiber supplementation, anorectal problems, abdominal pain, and history of opioid use in the last 48 h, any other factors which would interfere with constipation assessment and management, or pre-existing chronic diseases (such as severe heart, pituitary, thyroid, hematological, liver, renal, metabolic, neurological or mental diseases). Pregnant or nursing women were excluded. Women of childbearing potential were required to use effective birth control during the study. Noncompliant patients during baseline or treatment phases as evaluated by taking < 75% of either of the test articles during a one-week period throughout the course of the study and unable to provide informed consent were excluded from the patient data analysis. After they had provided written informed consent, participants were counselled at the initial visit to maintain their usual lifestyle, diet, physical activity, and diabetic treatment throughout the study. Participants were instructed to take 2 cookies (placebo cookies or flaxseed cookies) with a glass of water or tea twice a day at 10 am and 4 pm as a snack. The formulation of the flaxseed cookie was such that each cookie contained about 2.5 g of flaxseed. Thus, the 4 cookies per day consumed by each participant containing about 10 g of flaxseed. The stool diary was used to provide a stool accounting system and to obtain a subjective measure of efficacy. The participants were contacted at the end of week 1 to evaluate compliance to intervention. The clinician examined patients at baseline and each month after the start of therapy to evaluate the possible appearance of side effects of the interventions, and efficacy parameters.

The ROME III [13] definition was used for the chronic constipation by the presence of two or more of the following six complaints with at least 25% of bowel movements: straining, feeling of incomplete evacuation, hard or lumpy stool, feeling of anorectal obstruction/blockage, use of manual maneuvers, and less than 3 bowel movements/week.

### Randomization scheme

**Fig. 1:**
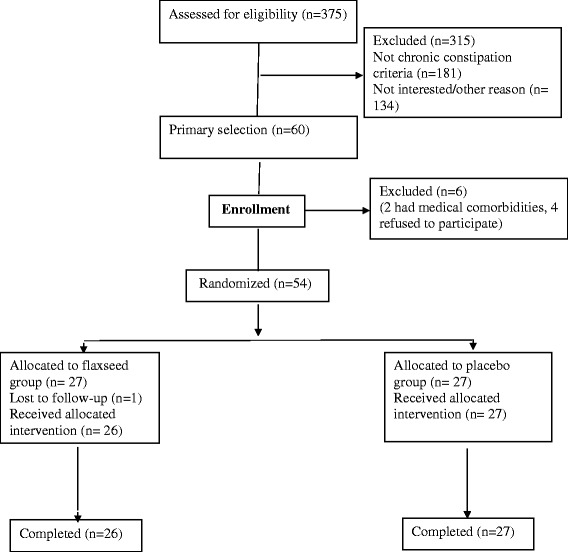
Design of the study

A total of 375 patients with T2D were screened and 60 patients were recruited. Four patients declined to participate, and 2 patients had medical comorbidities and did not meet our study criteria. The 54 participants (10 (18.5%) men, 44 (81.5%) women) were assigned randomly and equally to one of two treatment groups. Of those randomized, one patient in the flaxseed group lost to follow-up and were not evaluated (Fig. 1). Patients were randomized according to a preexisting list produced by a computer program that differed from a random number generator only in that it assigned equal numbers of patients to each treatment group, and the group assignments were concealed in an opaque sealed envelope.

### Intervention

Participants in the control group received sugar-free orange-flavored maltodextrin cookies twice per day for 12 weeks as placebo. The flaxseed group received 10 g flaxseed pre-mixed in a sugar-free orange-flavored maltodextrin cookies twice per day for 12 weeks. Each placebo cookie contained ~ 56.4 kcal and each flaxseed cookie contained ~ 59.8 kcal (Table 1). Participants were instructed to consume two cookies two times per day as a snack for a period of 12 weeks. A regimen of 4 cookies/ day was packed in individually labeled packs and provided to the individuals on a weekly basis. Cookie packs were labeled as cookie A and B. The study cookies was prepared by the Kamvar Co., Isfahan, Iran, who was not involved in patient care.

**Table 1:** Nutrient composition of placebo and flaxseed cookies

	Flaxseed cookie	Placebo cookie
Energy (kcal/cookie)	59.8	56.4
Carbohydrate (g/cookie)	7.4	8.1
Protein (g/cookie)	1.3	0.99
Fat (g/cookie)	3.1	2.0

The nutrient content was estimated using the Nutritionist IV dietary assessment software (Nutritional Database Manager 4.0.1, Nutritionist IV)

In order to assess the durability of flaxseed, constipation symptoms, glycemic, and lipid profile were assessed for another 4 weeks after stopping intervention, and the data from baseline, after 12 weeks of intervention, and the post-intervention periods were compared.

### Evaluation

The trial was single-blinded in that patients were blind to the treatment. Masking of the two treatments was preserved by creating cookies that looked, tasted, and textured identically. The differences in taste were minimal because the prominent flavor was that of the orange-flavor in which the cookies was mixed. The data were extracted and analyzed by one investigator (MJ) who was not involved with the study conduct. Only one author (NS) was not blinded to subject allocation and did not participate in data analysis.

### Measurements

All participants were 12-h overnight fast and data on age, gender, body size, education, fasting plasma glucose (FPG) (measured using the glucose oxidase method), glycosylated hemoglobin (HbA1c) (measured by ion-exchange chromatography), total cholesterol (TC), high-density lipoprotein cholesterol (HDLC), triglyceride (TG) (measured using standardized procedures), and low-density lipoprotein cholesterol (LDLC) (using the Friedewald equation [14]), were collected at baseline and at follow-ups. Height (assessed at baseline only) and weight (assessed at baseline and after the 12 week intervention) were measured in light indoor clothing without shoes to the nearest 0.5 cm and 0.1 kg, respectively. Body mass index (BMI) (kg/m^2^) was calculated as weight (kg) divided by the squared of height (m^2^). The physician defined T2D (as defined by the American Diabetes Association [15]) and all participants were under anti-diabetes medication for more than 3 years.

Participants in both groups maintained a constipation symptom diary for 12 weeks of intervention and for 1 week run-in phase and a 4-week without treatment. Constipation was assessed with a 5-point Likert Scale (not at all to all of the time) included bowel movement frequency, feelings of complete evacuation, use of digital maneuvers, stool consistency (Bristol Stool Form Scale), straining during bowel movement, pain during bowel movement, and overall feeling of constipation by a previously validated stool and symptom diary [16]. In addition, at the end of 4, 8, 12 and 16 week, patients were asked to fill out a global constipation symptom score. This validated Rome III outcome measure rated current constipation-related symptoms on a seven-point Likert scale (− 3 = markedly worse, − 2 = somewhat worse, − 1 = a little bit worse, 0 = no change, + 1 = a little better, + 2 = somewhat better, + 3 = markedly better) when compared to baseline symptoms. In the present study, the constipation symptoms evaluated by the Rome III criteria was considered as a continuous variable. The highest scores were indicative of the lower constipation symptom. The Bristol Stool Form Scale describes graphically the consistency and form of the stools in 7 categories: (1) nut likes; (2) lumpy sausage; (3) sausage with cracks; (4) smooth snake (adequate form); (5) soft blobs; (6) fluffy pieces; and (7) watery. Patients were instructed to record daily the stool consistency according to the Bristol Stool Form Scale. Participants were asked about any gastrointestinal disturbances or physiological changes. At the end of 12-week treatment period, patients were also asked to rate the looks, taste, and texture of the cookies that they had consumed on a visual analogue scale (0 = worst, 10 = best).

### Statistical analysis

Primary outcome measures included analysis of numerical values of constipation intensity according to global constipation symptom score at the beginning, 4, 8, 12 and 16 weeks after entry in flaxseed and placebo groups. Secondary outcome measures included analysis of the body weight, glycemic, and lipid control in the beginning, 4, 8, 12 and 16 weeks after entry in both groups. The sample size was calculated when the study was designed and was based on the comparison of two means. Assuming an SD for the treatment differences in global constipation symptom score of 2.5, as observed in other studies [17], we calculated that 27 patients per treatment group would be required to provide the study with 80% power to detect (with a two-sided alpha of 0.05) a mean difference in global constipation symptom scores of at least 1.5 between patients who received flaxseed vs. those who received the placebo. Statistical analysis was based on an intention-to-treat principle. The results for the groups that received flaxseed or placebo were compared with student’s t-test for independent samples; comparisons between basal and post-treatment periods were done by analysis of variance with repeated measures over time with a post hoc Bonferroni test. The sphericity assumption, which is required for the validity of repeated measure ANOVA was tested using Mauchly’s criteria, and when the sphericity assumption was not met, the Huynh-Feldt-Lecoutre Epsilon correction was used for *p*-values. Paired-Student’s t-test was conducted to analyze the difference between the baseline and week 12 for each of the two treatment groups, flaxseed and placebo. We used the chi-squared or Fisher’s exact test to compare proportions. To adjust for baseline characteristics in the analysis to account for the imbalance of the two groups, a general linear model was used to compare age, gender, LDLC, and constipation symptoms-adjusted means. The results are expressed as the mean (SD), and *P* < 0.05 was considered statistically significant. All statistical tests were two-sided, and all analyses were done with SPSS software for Windows (SPSS Inc., Chicago, IL).

## Results

### Characteristics

All 53 (flaxseed =26; placebo = 27) patients who completed treatment were available for follow-up at 4, 8, 12, and 16 weeks. Except for the higher LDLC and some constipation symptoms in the flaxseed group than in the placebo group at baseline, the two treatment groups were generally matched with regard to age, gender, diabetes duration, weight, BMI, FPG, HbA1c, triglyceride, HDLC, and constipation symptom score (Tables 2 and 3). Patients had mean (SD) duration of diabetes 8.5 (4.6) (flaxseed 8.7 (3.9), Placebo 8.3 (5.3)) years and mean age of 56.8 (8.0) (38.0 to 72.0) years at initial registration. Women accounted for 44 (83.0%), while men accounted for 9 (17.0%) of the 53 patients. Flaxseed cookies were well tolerated, with no serious adverse events.

**Table 2:** Characteristics of patients with type 2 diabetes and chronic constipation by treatment group at baseline

Characteristics	Flaxseed	Placebo	*P* value
	Mean (SD)	Mean (SD)	
Number of patients	26	27	–
Age (years)	55.7 (11.6)	55.9 (8.7)	0.955
Years since diabetes diagnosis	8.7 (3.9)	8.3 (5.3)	0.803
	No. (%)		
Constipation symptoms			
Straining	17 (65.4)	9 (33.3)	0.032
Hard stool	22 (84.6)	14 (51.9)	0.049
Pain with bowel movement	13 (50.0)	2 (7.4)	0.004
Feeling of incomplete evacuation	15 (57.7)	7 (25.9)	0.059
Digital maneuver	4 (15.4)	1 (3.7)	0.427
Fleeing of blockage	10 (38.5)	6 (22.2)	0.285
< 3 bowel movements/week	18 (69.2)	10 (37.0)	0.070
Therapeutic regimen			
Insulin	11 (42.3)	11 (40.7)	0.908
Metformin	21 (80.8)	20 (74.1)	0.560
Glibenclamide	3 (11.5)	8 (29.6)	0.104
Losartan	12 (46.2)	8 (29.6)	0.215
Metoral	2 (7.7)	1 (3.9)	0.530
Aspirin	11 (42.3)	8 (29.6)	0.336
Statin	12 (46.2)	15 (55.6)	0.494
BMI (kg/m^2^)			
Normal (BMI < 25)	4 (15.4)	8 (29.6)	0.396
Overweight (BMI 25–29.9)	12 (46.2)	12 (44.4)	–
Obese (BMI ≥ 30)	10 (38.5)	7 (25.9)	–
Gender			
Male	4 (15.4)	5 (18.5)	0.525
Female	22 (84.6)	22 (81.5)	–

*LDLC* low-density lipoprotein cholesterol, *HDLC* high-density lipoprotein cholesterol, *BMI* body mass index

**Table 3:** Comparison of constipation symptom score, weight, glycemic and lipid control in 53 patients with type 2 diabetes and chronic constipation before and after treatment with flaxseed and placebo

Variable	Treatment group		*P* value**
	Flaxseed	Placebo	
	Mean (SD)	Mean (SD)	
Global constipation symptom scores			
Baseline	−1.38 (0.8)	−1.00 (1.4)	0.627
After 4 week therapy	−1.77 (1.5)	− 1.74 (0.8)	0.257
After 8 week therapy	−0.50 (1.7)	−1.76 (0.8)	0.016
After 12 week therapy	1.08 (1.4)	−0.59 (1.4)	0.003
P value*	< 0.001	0.003	–
Change from baseline	2.46	0.41	0.008
After 4 week without therapy	−0.42 (1.6)	−0.81 (1.5)	0.257
Bristol Stool Form Scale			
Baseline	1.35 (0.5)	1.26 (0.5)	0.975
After 4 week therapy	2.38 (0.6)	1.59 (1.2)	0.026
After 8 week therapy	2.96 (0.6)	1.33 (0.6)	< 0.001
After 12 week therapy	3.31 (0.7)	1.85 (1.6)	0.005
P value*	< 0.001	0.307	–
Change from baseline	1.96	0.59	0.005
After 4 week without therapy	1.92 (0.8)	1.44 (0.8)	0.057
Weight (kg)			
Baseline	75.4 (10.6)	73.1 (12.0)	0.578
After 12 week therapy	71.6 (8.8)	73.1 (11.3)	0.749
P value*	< 0.001	0.947	–
Change from baseline	−3.8	0.0	< 0.001
Body mass index (kg/m^2^)			
Baseline*	29.1 (3.8)	28.7 (5.9)	0.976
After 12 week therapy	27.6 (3.1)	28.6 (5.6)	0.375
P value	< 0.001	0.930	–
Change from baseline	−1.5	−0.1	< 0.001
FPG (mg/dl)			
Baseline	164.8 (45.2)	165.6 (43.5)	0.980
After 4 week therapy	163.8 (45.1)	166.0 (42.5)	0.914
After 8 week therapy	143.4 (34.2)	168.0 (38.5)	0.016
After 12 week therapy	137.0 (26.4)	163.7 (39.5)	0.006
P value*	0.018	0.483	–
Change from baseline	−26.7	−1.9	0.011
After 4 week without therapy	140.0 (26.5)	170.1 (50.3)	0.013
HbA1c (%)			
Baseline	8.4 (2.0)	8.0 (2.2)	0.239
After 4 week therapy	8.2 (1.8)	8.9 (1.4)	0.740
After 8 week therapy	7.7 (2.1)	8.6 (2.2)	0.675
After 12 week therapy	7.7 (2.0)	9.0 (2.2)	0.101
P value*	0.137	0.978	–
Change from baseline	−0.8	1.0	0.001
After 4 week without therapy	7.0 (1.5)	8.5 (1.2)	0.009
Cholesterol (mg/dl)			
Baseline	178.1 (31.2)	177.3 (33.7)	0.480
After 4 week therapy	160.8 (31.1)	171.2 (30.1)	0.137
After 8 week therapy	148.1 (31.9)	171.4 (37.8)	0.017
After 12 week therapy	141.2 (31.9)	166.9 (39.8)	0.014
P value*	< 0.001	0.311	–
Change from baseline	−37.3	−10.4	0.085
After 4 week without therapy	147.6 (29.7)	170.0 (34.3)	0.280
Triglyceride (mg/dl)			
Baseline	161.1 (39.1)	172.2 (81.2)	0.221
After 4 week therapy	154.0 (45.3)	168.0 (58.0)	0.117
After 8 week therapy	154.0 (48.8)	165.0 (56.6)	0.230
After 12 week therapy	148.8 (45.7)	164.3 (56.3)	0.092
P value*	0.045	0.045	–
Change from baseline	−10.4	−11.4	0.283
After 4 week without therapy	149.8 (40.1)	167.6 (58.3)	0.118
LDLC (mg/dl)			
Baseline	115.1 (22.4)	94.5 (23.1)	0.008
After 4 week therapy	106.8 (22.5)	92.7 (26.6)	0.243
After 8 week therapy	99.7 (21.1)	96.1 (27.6)	0.003
After 12 week therapy	94.1 (19.9)	90.6 (17.9)	0.046
P value*	< 0.001	0.482	–
Change from baseline	−21.0	−4.3	0.010
After 4 week without therapy	105.0 (24.6)	100.5 (31.4)	0.636
HDLC (mg/dl)			
Baseline	43.1 (8.8)	43.5 (7.2)	0.992
After 4 week therapy	44.2 (9.3)	41.1 (7.9)	0.054
After 8 week therapy	45.6 (9.2)	42.0 (6.1)	0.726
After 12 week therapy	49.1 (7.9)	41.9 (10.0)	0.300
P value*	0.316	0.607	–
Change from baseline	4.7	−4.4	0.011
After 4 week without therapy	42.5 (8.1)	40.7 (6.5)	0.969
Cholesterol/HDLC ratio (mg/dl)			
Baseline	4.3 (1.2)	4.2 (1.0)	0.771
After 4 week therapy	3.9 (1.3)	4.4 (1.3)	0.052
After 8 week therapy	3.4 (1.2)	4.2 (1.6)	0.220
After 12 week therapy	3.0 (0.8)	4.2 (1.2)	0.048
P value*	0.021	0.332	–
Change from baseline	−1.4	−0.2	0.333
After 4 week without therapy	3.6 (0.9)	4.5 (1.4)	0.636

*Within group comparison

**Comparison between flaxseed and placebo groups, adjusted for baseline age, gender, LDLC, and constipation symptoms

### Constipation

Within-group analysis showed a significant decrease in the mean of global constipation symptom score in both groups (*P* < 0.001 for flaxseed and *P* = 0.003 for placebo). Mean global constipation symptom score changes was different between flaxseed and the placebo group (2.46 vs. 0.41; *P* = 0.008) (Table 3).

On the global constipation symptom survey, 2 (7.4%) patients who received placebo cookies and 11 (42.3%) patients who received flaxseed reported improvement of symptoms and rated their improvement as at least somewhat better (+ 2) when compared to baseline symptoms. The mean (SD) global constipation symptom scores after 12 week treatment were − 0.59 (1.4) and 1.08 (1.4) for placebo and flaxseed, respectively (*P* = 0.003).

When comparing placebo vs. flaxseed the stool consistency were different at 4, 8, and 12 weeks (*P* < 0.05). Comparing week 12 vs. baseline for placebo and flaxseed, stool consistency in flaxseed groups improved (*P* < 0.001), but not in placebo group (*P* = 0.307).

### Body weight

A significant decrease in body weight and BMI observed in the flaxseed group (P < 0.001), whereas no significant change was observed in the placebo group (*P* = 0.947 and *P* = 0.930). Mean weight loss and BMI changes from baseline was different between the flaxseed and the placebo group (− 3.8 vs. 0.0 kg and − 1.5 vs. -0.1 kg/m^2^; P < 0.001) (Table 3).

### Glycemic control

FPG and HbA1c were reduced in the flaxseed group while no changes observed in the placebo group. In the flaxseed group, the HbA1c shows reduced about 0.8%, in which this reduction was not significant (*P* = 0.137). Mean FPG and HbA1c changes from baseline were different between flaxseed and the placebo group (− 26.7 vs. -1.9 mg/dl; *P* = 0.011 for FPG and − 0.8 vs. 1.0%; *P* = 0.001 for HbA1c).

### Lipid control

Cholesterol levels decreased throughout the study period in the flaxseed group (*P* < 0.001), but not in the placebo group (*P* = 0.311). Mean cholesterol changes from baseline were different between flaxseed and the placebo group (− 37.3 vs. -10.4 mg/dl; *P* = 0.085).

Although within-group analysis showed a significant decrease in the mean of triglyceride in both groups (*P* = 0.045), change from baseline were not different between the two groups (− 10.4 vs. -11.4 mg/dl; *P* = 0.283).

While LDLC was decreased in the flaxseed group (changes from baseline, 21.0 mg; P < 0.001), no changes observed in the placebo group (*P* = 0.482). Mean changes from baseline in LDLC differed between the two groups (− 21.0 vs. -4.3 mg/dl; *P* = 0.010).

While HDLC was increased in the flaxseed group (non-significant), no changes observed in the placebo group. Mean changes from baseline in HDLC differed between the two groups (4.7 vs. -4.4 mg/dl; *P* = 0.011).

Although within-group analysis showed a significant decrease in the mean of cholesterol/ HDLC ratio in flaxseed (*P* = 0.021), but not in the placebo group (*P* = 0.332), change from baseline were not different between the two groups (− 1.4 vs. -0.2 mg/dl; *P* = 0.333) (Table 3).

After 4-week no treatment phase, the beneficial effects of flaxseed for glycemic and lipid levels, but not for constipation symptoms, appear to persist.

We re-analyzed the data with the baseline covariates of one drop out case and as expected the results were comparable for both analyses.

Patients rated both flaxseed and placebo cookies as palatable, looks, and texture with mean taste, looks and texture scores of 7.4, 7.7, and 7.4 for flaxseed cookies and 8.1, 7.9, and 7.6 for placebo cookies respectively.

## Discussion

The results of this study show that consumption of 10 g of flaxseed baked in cookies daily for 12 weeks improved constipation symptoms, glycemic and lipid control as well as BMI and body weight. No study has evaluated the impact of flaxseed for constipation in constipated patients with T2D. The decreased constipation symptoms by flaxseed observed in this study is consistent with previous studies in healthy volunteers [18], hemodialysis patients [19], and in animal studies [20]. As far as we know, the present study is the first specifically designed to evaluate the effects of flaxseed cookies on constipation in constipated patients with T2D.

This study also revealed significant, clinically meaningful reduction of 1.5 kg/m^2^ BMI and 3.8 kg body weight when the flaxseed group at baseline was compared with the flaxseed group after 12 weeks therapy. These results are consistent with those of other studies in which the body weight and other anthropometric measurements were significantly improved [7, 21, 22]. This change in body weight might be achieved by high fiber content and stimulation of satiety hormone production that’s enhancing satiety [23]. Even slight reductions in weight can produce metabolic improvements. The improved glycemic and lipid control in the flaxseed group could be attributed to changes in body weight, since statistically significant weight loss was seen 12 weeks after therapy. 2–5% weight loss was linked to improvements in FPG, HbA1c, total cholesterol, triglyceride, HDLC, but not LDLC [24]. However, it appears that increase intake of soluble fiber in the flaxseed group has certainly contributed to the observed results. The effect of flaxseed consumption on glycemic and lipid control has been reported in a few studies [8–10]. Nonetheless, administering flaxseed lignan in rodents was shown to prevent and delay the development of diabetes [25]. Pan et al. [8] also reported that flaxseed lignan improved glycemic control in patients with T2D. Another study reported, 5 g of flaxseed gum per day for 3 months reduced total and LDL cholesterol in patients with T2D [10], although one could expect a more pronounced effect among diabetics with dyslipidemia. Kristensen et al. [9] in an intervention trial observed a lowering of both total-cholesterol and LDLC within just 7 days in young healthy adults with normal blood cholesterol concentrations. A meta-analysis on the effects of flaxseed on blood lipids showed that flaxseed consumption, lower both total and LDLC, whereas flaxseed oil does not, and the role of lignans is still controversial [6]. Thus, consistent with these results, this study provides evidence for the first time, to our knowledge, that flaxseed may have a favorable impact on constipation symptoms, as well as, body weight, glycemic and lipid control in constipated patients with T2D.

Effect of flaxseed on glycemic control which showed an improvement in both FPG and HbA1c, is clinically meaningful which is comparable to the effect of many medications that are used to treat T2D, such as long term metformin therapy in the Diabetes Prevention program [26].

Furthermore, we conducted a 4-week follow-up assessment and found that the beneficial effects of flaxseed for glycemic and lipid levels, but not for constipation symptoms, appear to persist even after 4 weeks, suggest a durable effect on glycemic and lipid levels, at least in the short term. The constipation symptoms returned to the pre-study baseline levels, suggesting that the improvements observed during the study were due to the treatments and not a placebo effect or observational bias.

One limitation of flaxseed consumption that may cause people to discontinue treatment include taste, texture of the drink and dissolvability in a solution. We used cookies and the participants scored the taste and texture of cookies as acceptable.

The mechanism of action of flaxseed in reducing constipation symptoms, body weight, glucose, and lipid levels in constipated patients with T2D remains unclear. Limited evidence suggests that the abundance of polyunsaturated fatty acid in the diet might serve as an important modulator for body fat deposition. In a small clinical trial, Summers et al. [21] reported that changing from a diet rich in saturated fatty acid to one abundant in polyunsaturated fatty acid resulted in changes abdominal fat distribution and improves insulin sensitivity. A cross-sectional study also reported that a high dietary polyunsaturated fatty acid: saturated fatty acid ratio was inversely associated with waist circumference and waist/hip ratio [27]. The most plausible mechanism of action of flaxseed in reducing lipid profiles is through an interference with bile acid metabolism, where an increased intraluminal viscosity can hinder micelle formation and thus diminishes lipid uptake and inhibit re-uptake of bile acids causing the increased hepatic synthesis of bile acids which diverts cholesterol away from lipoprotein synthesis in the liver, thereby reducing serum cholesterol [9, 28]. It is believed that gel-forming fibers improved glucose homostasis and lipid and lipoprotein profiles [29, 30], by increase the viscosity of chyme in the upper intestine which may reduce the contact with digestive enzymes and delays absorption. The fiber fermentation in the intestine produces short-chain fatty acids that have been shown to be effective in enhancing peripheral insulin sensitivity [30]. The fermentation of fiber may influence gut microbiota and the alterations of microbiota may be responsible for improved levels of systemic inflammatory cytokines [31].

Limitations of this trial include the single-blinded design, the short follow-up, and small sample size. Albeit the value of the double blinded, controlled trial is widely recognized, this design is not always appropriate or indicated and it is quite difficult to do blinded trials for a food intervention. A randomized, double-blinded trial would be ideal and would eliminate the placebo effect. The duration of this trial, although fairly typical for dietary interventions, may be relatively short for evaluating the impact of flaxseed. Whether the beneficial effects of this short-term flaxseed intake will persist in the longer term is not clear. It is possible that a 4-week post treatment follow-up may be short to appreciate the real impact of the therapy. Assessing the efficacy in the long-term period is therefore warranted. While the number of patients studied was small, the effect was robust. Although we recruited constipated patients with T2D from a single tertiary care center who fulfilled the Rome III criteria and most of the participants were women, generalizability to other populations is unknown. Because the evaluation of the constipation symptoms by the Rome III criteria is subjective, we cannot exclude a placebo effect because of their expectations of the treatment. In addition, although the patients were advised not to change their dietary patterns, we did not assess changes in food and fluid intake, which might have influenced the results. In this study, selection bias was controlled for by randomization with concealed treatment allocation and observation bias was probably marginal because clinical results were a single-blinded analysis. However, constipation symptoms at baseline slightly differed between the two groups; with the higher constipation symptoms in the flaxseed group (presumably a chance effect). So, it could be argued that the treatment group “regressed to the mean”. However, we adjusted for baseline covariates to account for the imbalance of the two groups.

Based on the partial therapeutic benefit obtained with flaxseed cookies, its ease of administration, and lack of major side-effects, these results suggest that flaxseed in the form of cookies is useful in the treatment of constipation symptoms as well as the control of weight, glycemia, and lipids in people with T2D. Accordingly; none of our studied patients had any complaints about the application of this therapy.

## Conclusion

In constipated patients with T2D, flaxseed consumption may relieve constipation symptoms as well as decrease body weight, glycemic and lipid levels. Patients with T2D should be encouraged to increase the fiber content of their diet. Flaxseed consumption may be a valuable dietary approach for the prevention and treatment of T2D. The encouraging results obtained in this trial highlight the need for a larger sample size and longer follow-up period, probably blinded, to confirm these findings and better understand the mechanisms by which flaxseed may relieve constipation in patients with T2D.

## Abbreviations

ANOVA: Analysis of variance
BMI: Body mass index
FPG: Fasting plasma glucose
HbA1c: Glycosylated hemoglobin
HDLC: High density lipoprotein cholesterol
LDLC: Low-density lipoprotein cholesterol
SD: Standard deviation
T2D: Type 2 diabetes
TC: Total cholesterol
TG: Triglyceride
